# Fine-scale processes shape ecosystem service provision by an Amazonian hyperdominant tree species

**DOI:** 10.1038/s41598-018-29886-6

**Published:** 2018-08-03

**Authors:** Evert Thomas, Rachel Atkinson, Chris Kettle

**Affiliations:** 1 Bioversity International, Lima, Peru; 2 Bioversity International, Rome, Italy; 3 ETH Zürich, Institute of Terrestrial Ecosystems, Ecosystem Management, Zürich, Switzerland

## Abstract

Conspecific distance and density-dependence is a key driver of tree diversity in natural forests, but the extent to which this process may influence ecosystem service provision is largely unknown. Drawing on a dataset of >135,000 trees from the Peruvian Amazon, we assessed its manifestation in biomass accumulation and seed production of Brazil nut (*Bertholletia excelsa*) which plays a keystone role in carbon sequestration and NTFP harvesting in Amazonia. For the first time, we find both negative and positive effects of conspecific proximity on seed production and above ground biomass at small and large nearest neighbour distances, respectively. Plausible explanations for negative effects at small distances are fine-scale genetic structuring and competition for shared resources, whereas positive effects at large distances are likely due to increasing pollen limitation and suboptimal growth conditions. Finally, findings suggest that most field plots in Amazonia used for estimating carbon storage are too small to account for distance and density-dependent effects and hence may be inadequate for measuring species-centric ecosystem services.

## Introduction

The importance of Amazon forests for the provision of ecosystem goods and services to human society is well recognized. Recent research has suggested that the production of ecosystem services by Amazonian trees is disproportionally delivered by a small number of hyperdominant species that are extremely common and abundant in one or more Amazonian forest region^1,2^. Two key traits underlying tropical tree species ability to provision ecosystem services are above ground biomass (AGB) and fruit production^1,3,4^. The reproductive output of tropical trees is essential for the maintenance of trophic dynamics, and the sustainability of extractive non-timber forest product (NTFP) economies, while standing tree biomass is a key trait for terrestrial carbon storage and consequently climate regulation and wood provisioning services, among others.

Carbon storage assessments in Amazonia are typically based on measuring AGB, either through the use of sample plots^5^, or the application of remote sensing techniques^6^. Both methods have limitations. One of the main flaws of remote sensing is that it does not take into account the known variation in tree wood density and allometry across species^7^. The use of forest plot level measurements for scaling up estimates of carbon storage is increasingly questioned for the inability of plots to account for spatial heterogeneity in ecosystem services^8^ and their high sensitivity to the distribution of natural disturbance events across the landscape^9^. Additionally, plot-based assessments of ecosystem services related to standing biomass and fruit production of individual tree species are likely to be affected by scale effects. This is particularly so because most forest plots are less than one hectare^1^ while most tree species in Amazonia, including many hyperdominant species, occur at densities below one tree per hectare^2^. At increasingly fine scale, there is a higher likelihood that plot-based measurements of species-centric AGB and seed production will be influenced by conspecific distance and density-dependent effects.

Conspecific distance and density dependence (DDD) in trees is pervasive. Probably the best-studied of these is negative DDD in tree recruitment^10,11^ commonly known as the Janzen^12^ - Connell^13^ hypothesis. This hypothesis posits that survival of seedlings and saplings will be lowered closer to conspecific adults or in neighborhoods with high conspecific densities that attract predators, herbivores and host-specific pathogens. However, DDD has also been found to persist beyond the seedling stage^14,15^ and influence survival^16–18^, growth^19^ and fecundity^20–22^ of adult trees. The ecological processes underlying such distance and density effects in adult trees are not limited to pest and disease pressure and can generate either negative or positive DDD.

According to the resource niche partitioning hypothesis negative DDD is expected for growth and survival of adult trees at higher conspecific densities or shorter conspecific distances owing to increased intraspecific competition for limited resources^23,24^. By contrast, seed production is generally expected to be higher in trees that grow closer to conspecific neighbours or in areas with higher conspecific density owing to higher pollination distances and increased selfing rates in more isolated trees^21^. Such positive DDD fecundity has been described for numerous tree species around the world^25–27^ and may be manifested particularly in predominantly cross-pollinated (self-incompatible) species which make up the large majority of tropical tree species^28^. However, there is growing evidence that positive density-dependent fecundity gains may be lowered^21,29^ or even neutralised^30^ by counteracting forces related with positive fine-scale spatial genetic structures within tree stands^31^. Particularly in species with limited seed dispersal there is a higher likelihood that trees at shorter distances to conspecific neighbours are more genetically related^32^. Crosses between individuals with high kinship result in elevated biparental inbreeding which may lead to an increase in seed abortion rates^33^, and hence lower the seed set^34^.

The potential influence of DDD on the generation of ecosystem services related with biomass and fruit production by Amazonian hyperdominants is unclear. A better understanding of these effects is important for the interpretation of plot-based ecosystem service studies, given that the the spatial scale at which DDD operates as reported in literature transcends the dimensions of most Amazonian plots^1,5^. Being one of the largest and most long-lived of all hyperdominant tree species in Amazonia^2,35,36^, Brazil nut (*Bertholletia excelsa*) presents an excellent test case. It not only plays a keystone role in the ecology and nutrient cycling of Amazonian forests^37^, but has supported human livelihoods ever since the peopling of the Amazon^38,39^. Brazil nut seed is one of the cornerstone non-timber forest products (NTFPs) in Amazonia, sustaining multimillion dollar extractive economies in Bolivia, Brazil and Peru^40^. The species’ pivotal role in carbon sequestration has also been recognized. In a study on biomass accumulation among 3,458 Amazonian tree species, Brazil nut scored third highest^1^.

Like most tropical tree species^41^, Brazil nut occurs in aggregated distribution patterns due to combined effects of short-distance seed dispersal by rodents^42,43^ and anthropogenic activities^38,39,44^. However, in Peru anthropogenic effects appear to have been minimal compared to central and eastern Amazonia^38^. Limited seed dispersal of Brazil nuts is expected to create positive fine-scale spatial genetic structures, i.e. neighbourhoods in which trees are surrounded by genetically more related individuals^45,46^. These effects are counteracted by the species’ mating system. Brazil nut has allogamous flowers which are pollinated by large bees (mainly *Bombus*, *Centris*, *Xylocopa*, *Epicharis* and *Eulaema* species) capable of flying long distances (>20 km), thus ensuring extensive gene flow between distant Brazil nut trees and populations^47,48^.

We assessed DDD effects on the seed production and aboveground biomass (AGB) of individual Brazil nut trees and evaluated their impact on the accuracy and precision of simulated sample plots of different sizes to measure expected area-based ecosystem service provision. In line with the literature, we expected to find negative DDD effects for AGB and positive to neutral DDD effects for seed production. Additionally, we investigated the impact of the spatial aggregation of a Brazil nut tree’s conspecific neighbourhood on its seed production and AGB. On the premise that fine-scale spatial aggregation is expected to be mainly controlled by natural seed dispersal processes, Brazil nut trees in highly aggregated stands might be expected to have higher kinship. Hence, we hypothesized that the degree of aggregation in a focal tree’s conspecific neighbourhood would have a stronger influence on its seed production than on its AGB.

Drawing on a dataset of >135,000 Brazil nut trees from concessions in the Peruvian Amazon of Madre de Dios we found evidence of negative effects of conspecific density and spatial aggregation on seed production and AGB. Distance-dependent effects were more nuanced and showed increasing average seed production and AGB of Brazil nut trees with increasing nearest conspecific neighbour distances in the intervals of roughly 0–90 and 0–170 m, respectively, after which both decreased again. Our findings show that the precision and accuracy of sample plots to predict expected area-based seed production and AGB increase asymptotically with plot size, and suggest that most currently used Amazonian plots may underestimate the ecosystem services of individual tree species.

## Methodology

### Data

We used georeferenced data collected from 135,528 Brazil nut trees which had diameter at breast height (DBH) ≥10 cm from 418 Brazil nut concessions in Madre de Dios, Peru with areas ranging between 16 and 4,575 ha (Supplementary Fig. S1). Most of the data were collected in the period 2003–2007, in response to the Peruvian Forestry Law N°27308 (5/10/2001), which obliged concession holders for the first time to present detailed inventories of the Brazil nut trees under their custody. Inventories were carried out by a number of institutions active in the region, notably ACCA (Asociación para la Conservación de la Cuenca Amazónica), CAMDE (Conservación Ambiental y Desarrollo en el Perú), FONDEBOSQUE (Fondo de Promoción del Desarrollo Forestal), AIDER (Asociación para la Investigación y Desarrollo Integral), RNTAMB PRMRFFS (Programa Regional de Manejo de Recursos Forestales y Fauna Silvestre), Forestal Rio Piedras SAC and Conservation International. Field staff from these institutions georeferenced all individual trees, asked concession holders who accompanied them to estimate the average productivity of each individual tree, and measured the DBH of most of the trees (114,994). The datasets analysed during the current study are available from the corresponding author upon request.

Brazil nut seeds are harvested by cracking open the lignified capsular fruits with a machete after they have fallen on the ground. Individual seeds are protected by an additional wooden testa (shell), but these are not opened in the field and harvesters express seed production weight in terms of ‘latas’ (tin cans) which contain approximately 11.66 kg of fresh in-shell seeds. Thomas *et al*.^49^ have shown that pooled seed production estimates by Brazil nut harvesters from Madre de Dios are accurate enough to generate valid statements about the relation between environmental, morphological and phytosanitary variables and seed production. Here we use these estimates as a proxy for the actual average seed production of Brazil nut trees. Human harvesting typically begins once nearly all fruits have fallen. Dispersers have been estimated to consume and disperse approximately 3% of seeds prior to this, and hence secondary dispersal across the forest floor is likely to only minimally bias productivity estimates by harvesters^50^. Seed production estimates for individual trees varied from 0 to 362 kg, with an average of 30.3 ± 26.9 (SD) kg per tree. We used the generalized equations from Chave *et al*.^51^ to calculate above ground biomass (AGB) of individual Brazil nut trees using an overall wood density value of 0.59 g cm^−3 ^^52^. AGB estimates varied from 18 kg to 121.90 tonnes per tree, with an average value of 10.25 ± 8.07 tonnes. The relationship between AGB and estimated seed production of individual trees was asymptotic to unimodal^49^. The concessions showed considerable variation in the average seed production (30.4 ± 11.6 kg), AGB (10.3 ± 3.3 tonnes) and density (0.53 ± 0.28 trees per hectare) of Brazil nut trees (Supplementary Fig. S2).

A limitation of this dataset is the low precision of georeferences of numerous individual trees^49^. However, these imprecisions are not expected to obscure trends for two reasons. First, our analyses are largely based on pooled results of spatial assessments within concessions, where spatial relations between trees are likely to be conserved. Second, the large size of our dataset is likely to compensate for possible noise introduced by imprecise spatial location data.

### Statistical analysis

**Figure 1:**
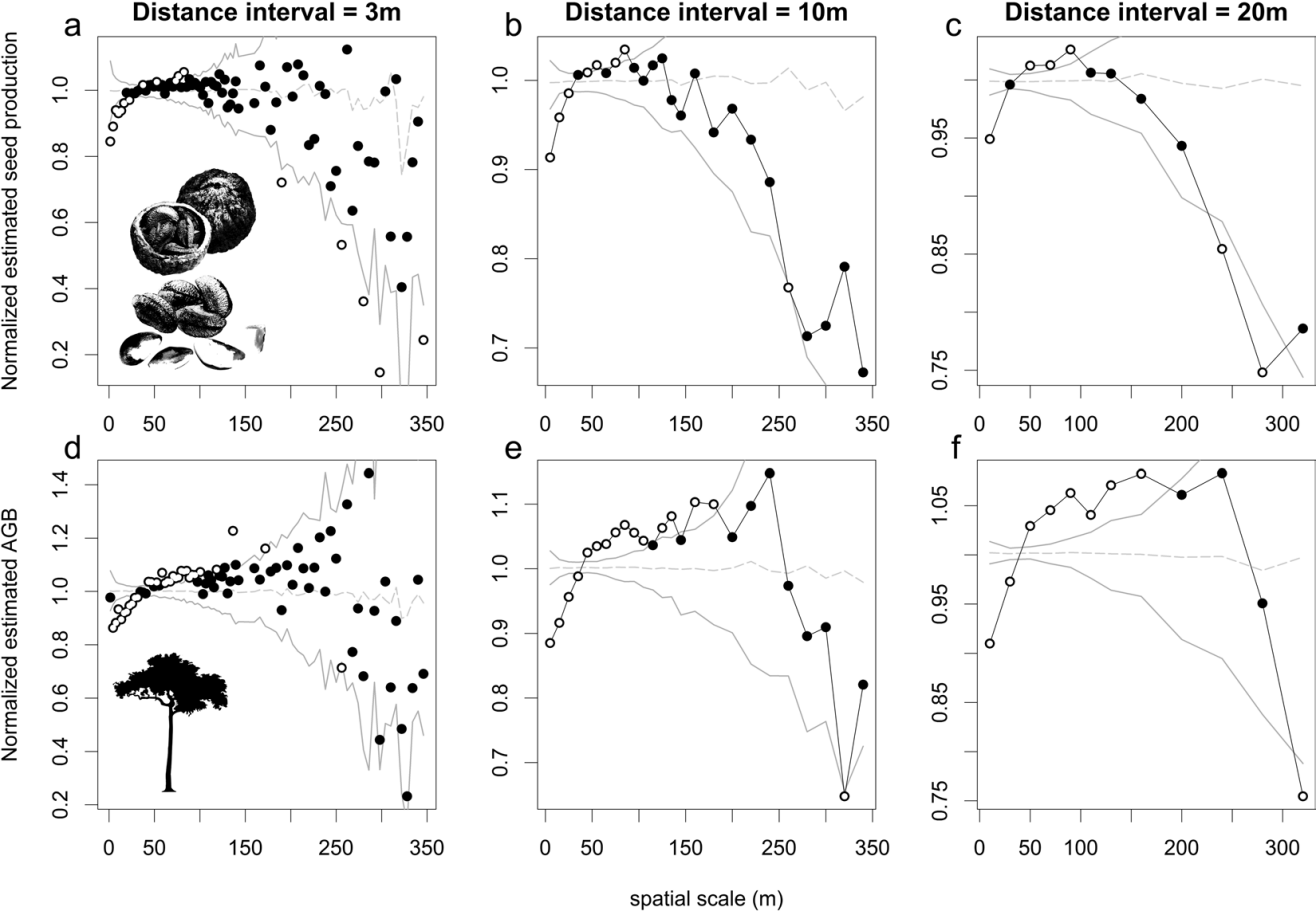
The first mark correlation function showing the mean normalized seed production (**a**–**c**) and AGB (**d**–**f**) of all Brazil nut trees located at distance r ± h from their nearest conspecific neighbours, for three different bandwidth and interval sizes (1.5, 5, 10 and 3, 10, 20, respectively). The mark correlation functions significantly depart from the null model (dashed grey lines) at particular distances r if their respective summary statistics of the observed data (dots) fall outside the simulation envelopes (solid grey lines). Significant departures from the null model are marked by white dots.

To assess the importance of DDD effects on estimated seed production and AGB we applied techniques of marked point pattern analysis, through the adaptation of the three mark correlation functions proposed by Fedriani *et al*.^30^ and one new one we put forward here. The first function represents the mean normalized estimated seed production or AGB of all individual Brazil nut trees with closest conspecific neighbours located within a given distance interval (r − h, r + h), where *h* is the bandwidth constructed around a distance class *r*. For normalization purposes, Fedriani *et al*.^30^ used the grand mean of plant reproductive success of all trees in their dataset. Where the study region of the latter authors consisted of one continuous 49 ha area, the concessions included in our study have a much wider spatial distribution. Therefore, we normalized seed production and AGB estimates of individual Brazil nut trees with the respective mean values of all trees from the same concession as the focal tree and used these normalized estimates to calculate mean values per distance interval. This neutralizes the differences in mean seed production and AGB estimates of Brazil nut trees across concessions (Fig. 1), which may be due to variation in local environmental conditions and/or differences in seed production estimates among harvesters. Hence, scores larger and smaller than 1 indicate that the estimated seed production or AGB of trees that have their nearest neighbours at distance r ± h is on average larger or smaller than the mean estimated seed production or AGB in the respective concession, indicating positive and negative effects of nearest conspecifics on seed production or AGB, respectively.

The second mark correlation function is based on Schlater *et al*.^53^ and characterizes the spatial covariance in estimated seed production or AGB of any two Brazil nut trees separated by distance r ± h, resulting in a summary statistic similar to Moran’s I. “Schlater’s correlation function” corresponds with the classical Pearson correlation coefficient between the estimated seed production or AGB of all possible pairs of Brazil nut trees separated by nearest neighbour distances of r ± h^30^. We used the seed production and AGB estimates normalized per concession for calculating Schlater’s correlation function, in line with the arguments given above. Therefore, this function indicates how similar the estimated seed production and AGB is of trees separated by nearest neighbour distances of r ± h within a given concession.

The bandwidth must be wide enough to yield a sufficient number of data points in each distance class *r* but small enough to reveal relevant biological detail^30,54^. We considered three different bandwidth scenarios (1.5, 5 and 10 m) to assess the consistency of patterns in the above mark correlation functions at different spatial scales, and simultaneously test whether precision issues with the spatial information of some trees perturb patterns observed at the smallest bandwidth. Proportionally many fewer Brazil nut trees in our dataset grew at distances >150 m from their nearest conspecific neighbours. Therefore, at distances of up to only 150 m we estimated the above mark correlation functions every 3, 10 and 20 meters, with distance intervals being centred on r = (1.5, 4.5, 6 …, 148.5), (5, 15, 25 …, 145 m) and (10, 20, 30, 140 m), respectively. For the stretch from 150 to 350 m, we used bandwidths of 3, 10 and 20 meters and intervals of 6, 20 and 40 m to increase the number of data points in each distance interval. However, this was still not sufficient to obtain a stable pattern for Schlater’s correlation function, for which only half as many data points are available as for the normalized estimated seed production and AGB functions. Therefore, we only show results for the first 210 m for this function.

The third and last mark correlation function proposed by Fedriani *et al*.^30^ is the Pearson correlation coefficient between the estimated seed production and AGB of a focal Brazil nut trees, and the number of its conspecific neighbours within the distance interval from 0 to r, called “density correlation function”. Following Fedriani *et al*.^30^, the distance intervals considered for this cumulative density correlation function were r = 10, 20, 30 …, 350 m.

Finally, we developed a mark correlation function that relates the estimated seed production and AGB of individual Brazil nut trees to the degree of spatial aggregation of its conspecific neighbours. This “aggregation correlation function” is the classical Pearson correlation coefficient between the estimated seed production and AGB of a focal tree and the degree of spatial aggregation of all Brazil nut trees within distance interval r of that tree, using Clark and Evan’s^55^ method. This method posits that the expected mean distance to the nearest neighbour equals $E(d)=\,\frac{\surd \rho }{2}$, where ρ is the known tree population density. Owing to the variation in Brazil nut densities across concessions, for calculating the spatial aggregation of the conspecific neighbours of each focal Brazil nut tree we used the average density value obtained for the respective concession in which it was located. An index of spatial aggregation was then calculated for the spatial neighbourhoods constructed around each focal tree as the ratio $\bar{{\rm{d}}}/{\rm{E}}({\rm{d}})=2\bar{{\rm{d}}}\sqrt{\rho }$, generating a value of 1 for random patterns, more than 1 for more even spacing patterns, and less than 1 for aggregated patterns^55^. Hence positive scores for this mark correlation function imply that the AGB or seed production of focal trees tend to be lower in conspecific neighborhoods with higher levels of spatial aggregation, while negative scores point to the opposite.

We corrected for edge effects in all the above mark correlation functions by excluding trees from the respective analyses whose distances to the closest edge of concessions were shorter than to their closest conspecific neighbours (first two functions; 4% of the trees dropped from the analyses) or shorter than the radius *r* of the circular neighbourhoods constructed around them (last two functions). For the fourth function, we additionally removed conspecifics from spatial randomness calculations whose distances to the circumference of the circular neighbourhood with radius *r* constructed around each focal tree were shorter than to their closest conspecific neighbours.

To test the statistical significance of spatial trends in the four mark-correlation functions, we compared our results with those of spatial correlation-free null models. The null models were implemented by randomly shuffling the seed production and AGB estimates of all individual Brazil nut trees, following Fedriani *et al*.^30^ and references therein. We carried out 199 randomizations to construct simulation envelopes for the summary statistics of each of the mark correlation functions, corresponding with the fifth lowest and highest values (2.5 and 97.5th percentiles) of the summary statistics. The summary statistic of the observed data at particular distances *r* significantly departs from the null model if it is outside the simulation envelopes. All marked point pattern analysis were carried out with custom-made scripts in R^56^ using packages *maptools*^57^ and *spatstat*^58^. R codes are available from the corresponding author on request.

To test the precision and accuracy of different plot sizes to predict expected area-based estimated seed production and AGB of Brazil nut, and validate the results of the mark functions, we generated maps of all variables by projecting the trees in our dataset on raster maps with different grid cell sizes (1; 2.5; 3.33; 5; 7.5; 10; 15; 20; 30 and 40 arc seconds) and converted these to hectare-based surface values. Density maps were created in *raster* package for R^59^ by counting the number of trees per grid cell. Maps of spatial randomness were generated through application of Clark and Evan’s^55^ method and correction of edge effects by excluding trees that were located closer to a grid cell edge than to their closest neighbours from average nearest neighbour distance calculations per grid cell. Raster maps of expected and measured estimated ABG and seed production were obtained by multiplying the number of trees per grid cell with the grand mean estimated AGB and seed production, respectively, and summing the corresponding values of individual trees per grid cell. We used linear regression to assess the accuracy and precision (expressed by the regression slope and R^2^, respectively) of different grid cell resolutions to predict expected area-based seed production and AGB of Brazil nut. Model residuals did not show evidence of spatial autocorrelation as confirmed by means of autocorrellograms constructed in *ncf* package for R^60^.

## Results

### Mark correlation functions

The first three mark correlation functions yielded remarkably similar trends for the estimated seed production and AGB of Brazil nut trees and patterns in the first two functions were conserved across the different bandwidths tested, suggesting precision issues with some of the georeferences did not perturb the findings. The estimated seed production and AGB were significantly lower than average for Brazil nut trees whose closest conspecific neighbours were located at distances below 30 and 40 m, respectively (Fig. 1). By contrast, at intermediate conspecific neighbour distances most scores were significantly higher than average, but this distance interval was narrower for estimated seed production (50–90 m) than AGB (50–170 m). At distances beyond 150 and 250 m estimated seed production and AGB, respectively, showed steep decreases. While at the smallest bandwidth (Fig. 1a,d) most scores did not differ significantly from the overall means, at larger bandwidths (Fig. 1b,c,e,f) significantly lower scores were found beyond 250 and 300 for seed production and AGB, respectively. We found very similar unimodal relationships as in Fig. 1 between the raw estimated seed production and AGB of a given Brazil nut tree and the distance to its first five nearest conspecific neighbours (Supplementary Figs S3,4).

**Figure 2:**
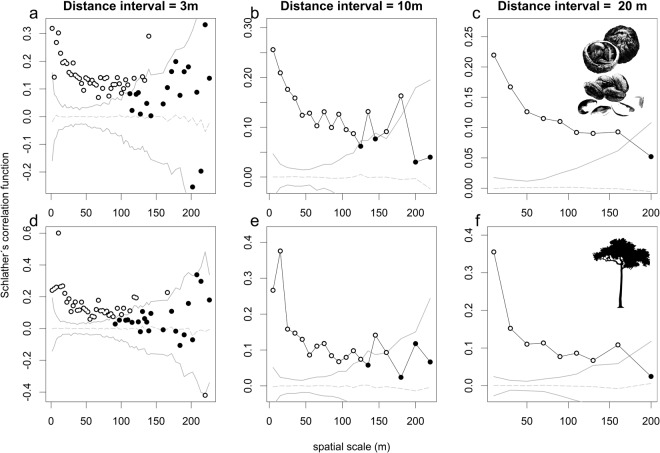
Schlather’s mark correlation function, representing the correlation between the estimated seed production (**a**–**c**) and AGB (**d**–**f**) of any pair of Brazil nut trees separated by nearest neighbour distances r ± h, for three different bandwidth and interval sizes (1.5, 5, 10 and 3, 10, 20, respectively). The mark correlation functions significantly depart from the null model (dashed grey lines) at particular distances r if their respective summary statistics of the observed data (dots) fall outside the simulation envelopes (solid grey lines). Significant departures from the null model are marked by white dots.

**Figure 3:**
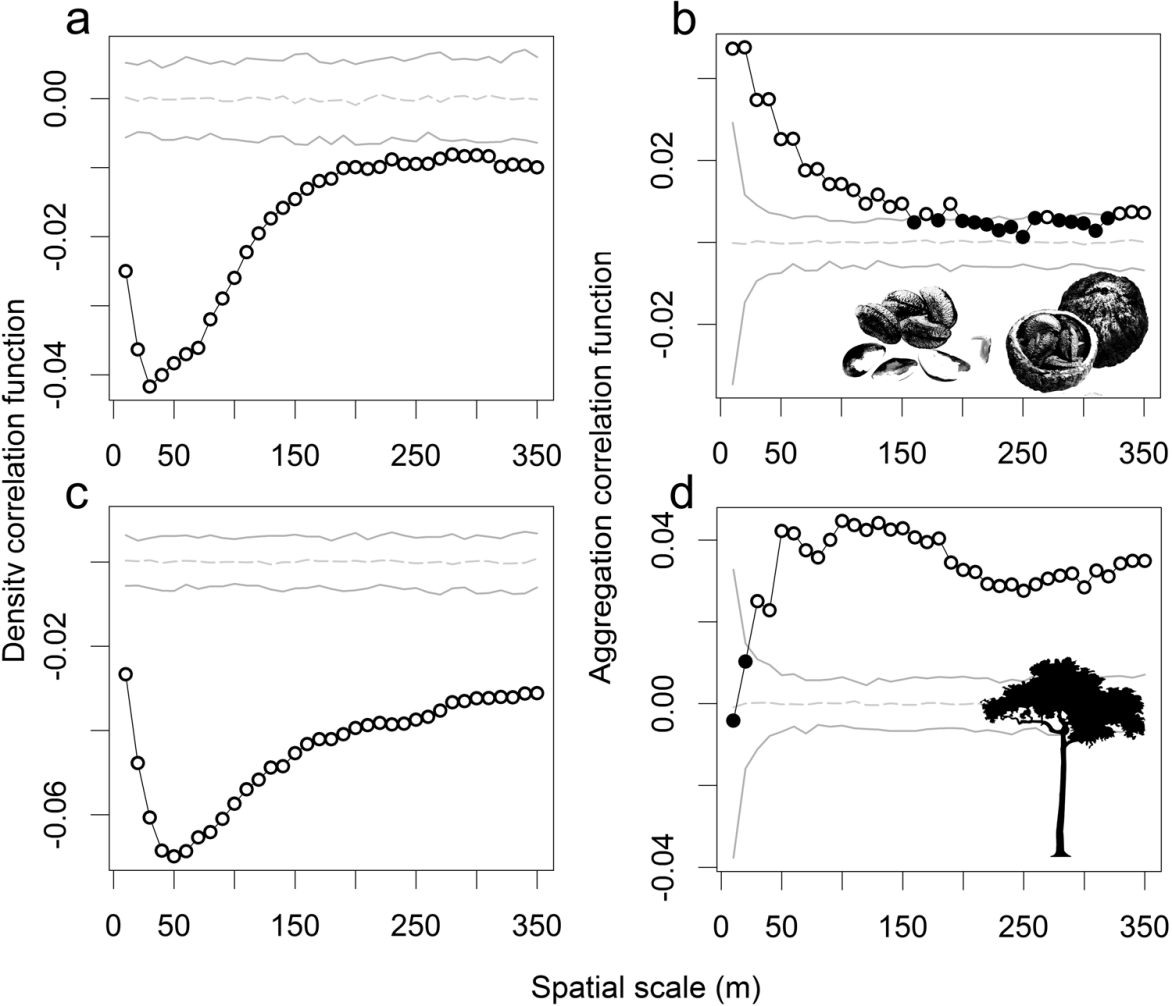
The density and aggregation correlation functions, representing the correlation between the estimated seed production (**a**,**b**) and AGB (**c**,**d**) of a focal Brazil nut tree and the number and degree of spatial aggregation of conspecific neighbours in the circular neighbourhood with radius r constructed around the focal tree, whereby r = 10, 20, 30 … 350. The mark correlation functions significantly depart from the null model (dashed grey lines) at particular distances r if their respective summary statistics of the observed data (dots) fall outside the simulation envelopes (solid grey lines). Significant departures from the null model are marked by white dots.

Schlather’s correlation function showed that the estimated seed production and AGB of pairs of nearest neighbours separated by less than approximately 150 m were positively correlated (Fig. 2). The density correlation function indicated a highly significant negative density dependence in circular neighbourhoods up to 150 m and beyond 350 m diameter for a tree’s estimated seed production and AGB, respectively, with the strongest correlations occurring between 20 and 100 m (Fig. 3a,c).

Finally, the aggregation correlation functions showed that a focal tree’s estimated seed production and AGB tended to be lower in neighbourhoods with higher levels of spatial aggregation, i.e. negative spatial aggregation dependence (Fig. 3b,d). However, at small neighbourhood sizes the trends differed between estimated seed production and AGB. For seed production, correlation coefficients were highest for the smallest neighbourhoods, and decreased with increasing neighbourhood size to become insignificant for circular neighborhoods with diameters beyond 250 m. By contrast for AGB, correlation coefficients were not significantly different from the null model at the smallest neighbourhood sizes, and reached their maximum value in neighbourhoods of 50 m radius and beyond. This suggests that in small neighbourhoods the AGB of focal trees is only influenced by the number of conspecific neighbours, but not their spatial arrangement, while the estimated seed production tends to be lower in both denser and more aggregated conspecific neighbourhoods, in line with the initial hypothesis.

Most Brazil nut trees in our study area tended to occur in an aggregated pattern (spatial aggregation scores <1; Fig S5). Spatial aggregation was a poor predictor of stem density, area-based AGB and seed production estimates (Supplementary Figs S5–7), but the highest values of all three variables tended to be found at intermediate levels of spatial aggregation.

### Area-based assessments of Brazil nut ecosystem services

**Figure 4:**
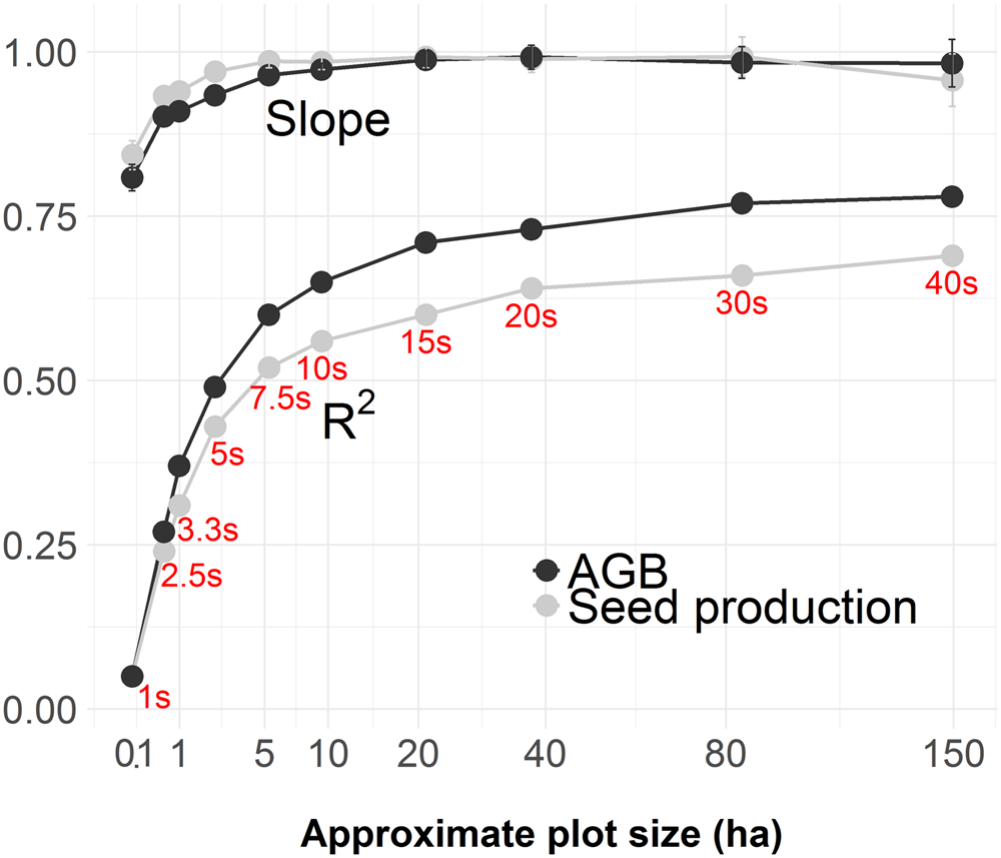
Slope and R^2^ of linear regressions between expected and observed AGB and estimates of seed production in simulated evaluation plots of different sizes plotted on a square root scale. The corresponding grid cell sizes (arc seconds) of the raster maps used for generating the graphs and conversion to hectare based surface values are shown in red. Error bars for slope values are 95% confidence intervals.

The slope (proxy for accuracy) and R^2^ (proxy for precision) of linear regressions between expected and measured area-based estimates of seed production and AGB of Brazil nut increased asymptotically with increasing grid cell size (Fig. 4 and Supplementary Figs S8,9). The asymptotes start levelling off around 5 ha at which point regression slopes are close to 1 and the R^2^ reaches 0.5–0.6. R^2^ values remained well below 1 at different spatial resolutions which is likely partly due to the environmental variability across the research area which implies that grand means of AGB and seed production can over- or underestimate expected values depending on the locations of grid cells.

## Discussion

Our analyses indicate that DDD responses of traits which are important for the provision of ecosystem services by Brazil nut operate at different spatial scales. At fine spatial scales (<40 m and <50 m), proximity to conspecifics had a negatively effect on seed production and AGB of focal Brazil nut trees, while at larger nearest neighbour distances (>150 m and >250 m, respectively) positive effects were apparent. We posit that each of these trends may have different underlying factors. Negative effects are likely caused by fine-scale genetic structuring and higher competition for shared resources at short distances, whereas positive effects of proximity in trees growing at longer nearest neighbour distances are likely due to pollen limitation and suboptimal growth conditions, respectively. These patterns are expected to significantly lower the accuracy and precision of area-based assessments of AGB and fruit production of Brazil nut when field plot sizes below 5 hectares are used, a pattern that could also apply to other hyperdominant tree species. Our findings suggest that ubiquitous patterns of negative DDD of tropical tree recruitment^61^, including Brazil nut^62^, might be paralleled by similar effects on seed production and AGB. A limitation of our dataset is that we worked with seed production estimates instead of actual measurements. The correlation between estimates and two-year-average measurements yielded an R^2^ of up to 0.4^49^. While differences in units of measurements (multiples of 11.66 kg tin cans vs hectograms) and the fact that reliable tree-based productivity averages require multiyear measurements^63^ are likely to account for part of the unexplained variation, imprecisions and inaccuracies in productivity estimates are an additional source of bias. In spite of this, the fact that our data allowed detection of fine- and coarse scale distance, density and aggregation-dependent effects supports the notion that crowdsourced and citizen science data can offer a valuable alternative to costly field measurements^49^.

### Do trees with higher local conspecific tree density have lower fruit production?

Positive DDD effects on seed production in trees have been reported frequently^29,30,64^. Pollinators tend to maximize their reward while minimizing energetic expenditures^65^ and hence are attracted more to groups of conspecifics than to isolated trees. Furthermore, more isolated trees have a higher chance of receiving more heterospecific and less conspecific pollen due to more generalist than specialist pollinator visits^66,67^. Accordingly, Wadt *et al*.^45^ found that Brazil nut trees growing at larger conspecific distances in pastures were visited by lower numbers of pollen donors compared to trees growing in a forest matrix. More isolated trees are also more likely to suffer from higher rates of within-plant pollen movement, i.e. geitonogamy, which either enhances pollination failure due to incompatibility, or results in lower fruit set and seed viability^33,64^. Brazil nut is predominantly outcrossing^68^ and likely to be partially self-incompatible, thus preventing fruit set through self-fertilization^45^. In a hand pollination experiment, Cavalcante *et al*.^69^ showed that self-pollinated Brazil nut flowers did not produce any fruits.

Negative fine-scale effects of proximity on fecundity seem to have gone largely unnoticed until recently when scholars started taking into consideration the genetic relatedness of conspecific neighbours^21,29,30^. These studies confirmed previous findings of population geneticists^32^ that tree species with limited seed dispersal tend to enhance short-distance crowding of genetically related individuals. Conspecific neighbourhoods with high local kinship have been found to either lower the mean fruit set of individual trees^21^ or increase within-tree variance in seed viability^29^, due to an increased likelihood of biparental inbreeding. Biparental inbreeding can increase the frequency of seed abortion through early-acting inbreeding^33^, among others because embryos homozygous for deleterious alleles tend to die during development^70^. Additionally, related individuals may share incompatibility alleles that prevent the formation of embryos to begin with^71,72^.

Similar mechanisms are likely to explain at leat part of the fine-scale negative effects of proximity to conspecifics on seed production in Brazil nut. Baldoni *et al*.^46^ found positive genetic structures up to 175 m in two different areas in Brazil, one of which (Acre) is adjacent to our study region. While partial self-incompatibility in Brazil nut is likely to prevent self-fertilization, it does not seem to impede crossing among relatives^45^. This suggests that biparental inbreeding and not incompatibility may be the main mechanism underlying the significantly lower seed production of Brazil nut trees with nearest conspecific neighbour distances below 30 m. The fact that fruit production has been found to decrease in Brazil nut plantations established with genetically related trees^73,74^ supports this hypothesis.

Patterns of negative density-dependent fecundity as suggested here have also been reported for the understory palm *Geonoma epetiolata*^75^ and the pioneer tree *Cecropia obtusifolia*^20^, but do not seem to be universal. Several studies did find evidence of negative effects on fruit production due to higher kinship in denser conspecific neighbourhoods but these were not strong enough to compensate for the positive effects on fecundity of increased crosspollination^21,29,30^. The available studies in Neotropical species suggest that the nature of density-dependent fecundity might be related to the idiosyncrasies of each species’ reproductive biology. Positive density effects tend to be found in species with wind,^21^ bird, or bat-dispersed seeds^29^, which can be more effective in long distance dispersal than in species where dispersal is largely controlled by gravity^75^, or by mammals with very short dispersal ranges such as Brazil nut. Stronger fine-scale population genetic structures are expected in species with limited seed dispersal, leading to stronger negative fine-scale distance-dependent fecundity. This, in turn, might counteract the positive effects of increased cross-pollination in denser conspecific neighbourhoods, and result in net negative density-dependent fruit and seed production. As such, the fact that seed production of focal Brazil nut trees was more strongly negatively impacted by the spatial aggregation of conspecifics in smaller neighbourhoods than AGB, might indicate that fine-scale spatial aggregation (and genetic structure) in Brazil nut is mainly controlled by short-distance seed dispersal processes by scatter-hoarding rodents^42,43^. Further research is needed to further test the validity of these hypotheses.

While the genetic relatedness of Brazil nut trees growing close together presents a plausible explanation for the fine-scale negative distance dependence of seed production, there might be other causal factors that require further research. First, negative effects may -at least partly- be due to resource limitation^76^. The longstanding extraction of Brazil nut seeds from concessions may have resulted in local depletion of certain soil nutrients and hence lower average seed production in denser or more aggregated conspecific neighborhoods owing to increased competition for these nutrients. Second, spatial aggregation in Brazil nut might be due partly to contagious dispersal and heterogeneity in environmental conditions (soil nutrients, light availability). It is not uncommon that microsites suitable for recruits are not favorable for adults^77^ and this mismatch between requirements could be reflected in clumps of trees (due to high seedling establishment) showing proportionally lower performance at advanced ontogenetic stages. Third, although not well-understood, kin recognition might play a role too. Kin recognition has been recognized to enhance competition^78^ and cooperation^79^ among relatives in plants. It might, therefore, also play a role on early recruitment of certain genotypes with potential delayed consequences for performance.

### Do trees with higher local conspecific tree density have lower AGB?

The positive distance dependence of AGB in Brazil nut that we found at larger conspecific distances is intriguing and might be an expression of the fact that more isolated trees have a higher chance of growing in suboptimal growth sites, thus resulting in lower average AGB values. This is in line with the notion that species’ abundances decline with distance from the centroid of the species’ habitable conditions in environmental space (the ecological niche)^80^.

The negative distance-dependent AGB of individual Brazil nut trees we found at short distances is in line with the resource niche partitioning hypothesis^23,24^ which results in stronger intraspecific than interspecific competition for shared resources^14^. Higher conspecific densities or aggregations of trees have been found to correlate with lower growth rates and survival of individual trees^15,16,19,20,81^. An alternative hypothesis is that conspecific tree proximity, density or neighbour size might strengthen negative Janzen-Connell type effects of pathogens and insects on the survival and growth of adult trees^82^, in parallel with similar effects on seedlings^83^. Denser, more aggregated conspecific neighbourhoods and larger conspecific adult trees have had more time to accumulate natural enemies, which might result in slower growth or higher mortality of the trees growing in such neighbourhoods or closer to larger conspecifics, or lower the chances of new recruits in transitioning to larger diameter classes. If this hypothesis is correct, we might expect to find larger trees at sites with lower conspecific densities. The lack of spatial attraction between Brazil nut juveniles and adults recently documented in our research area^84^ supports this possibility. There is evidence that for a tropical tree, the chance of survival generally increases when it is surrounded by higher proportions of conspecific neighbours that are smaller^17^. While plant size has been found to positively influence the richness of above-ground enemies^82^, there are indications that soil-borne pathogens may play a more important role in negative size-dependent effects, possibly through the more extensive root systems of larger trees (Packer & Clay^86^; Mangan *et al*.^85^; Xu *et al*.^87^; Bachelot *et al*.^82^; but see Gilbert *et al*.)^88,89^. Further research is needed to test this hypothesis.

### Implications for area-based assessments of ecosystem service provision by tropical trees

Our findings suggest that the accuracy and precision of area-based appraisals of ecosystem service production by tropical tree species that rely on tree measurements can be greatly influenced by field plot size. Only at spatial resolutions above 5 ha was the correspondence between expected and measured AGB and seed production estimates more than 50%. Smaller plot sizes yield less precise measurements, and underestimate area-based seed production and AGB (Fig. 4), owing to the progressive manifestation of border effects related with negative distance, density and aggregation dependence. The large majority of field plots in Amazonia have sizes of 1 ha or smaller, such as the 0.1 ha sample units included in the Alwyn H. Gentry Forest Transect Dataset^1,2^. This suggests that most current field plots may be inadequate for measuring species-centric ecosystem service provision in Amazonia.

## Conclusion

Many Amazonian hyperdominant tree species not only share similar spatial distributions (local occurrence at high densities and in clumped distributions) and reproductive traits (short dispersal distances) as the Brazil nut, but also play dominant roles in the forest’s ecosystem service provision (carbon cycling, fruits used as NTFPs etc.)^1,2,90^. This implies that negative DDD of tree biomass accumulation and fecundity, and associated ecosystem services, might be pervasive in Amazonia. Furthermore, the effect of negative DDD on the recruitment of rare species, which occur in low to very low population sizes and densities but make up the vast majority of tree species in Amazonia^2^, is known to be much stronger than for common species^61^. The extent to which these patterns are paralleled by stronger negative DDD on seed production and AGB is currently unknown. Testing the hypotheses we put forward here for Brazil nut is needed to establish the broader relevance of these processes for shaping ecosystem service provision for Amazonian hyperdominants and rare species.

## Electronic supplementary material

Supplementary Information 1
