# Supplementary material for: Fine-scale processes shape ecosystem service provision by an Amazonian hyperdominant tree species
Source: Sci Rep. 2018 Aug 3;8:11690. doi: 10.1038/s41598-018-29886-6 (PMC6076282; doi:10.1038/s41598-018-29886-6)
Supplement: Supplementary file 1 — Supplementary Information 1 [file 41598_2018_29886_MOESM1_ESM.docx]

**-Supporting Information-**

**Fine scale processes shape ecosystem service provision by an Amazonian hyperdominant tree species**

**Evert Thomas^1,*^, Rachel Atkinson^1^, Chris Kettle^2,3^**

^1^ Bioversity International, Lima, Peru, [evert.thomas@gmail.com](mailto:evert.thomas@gmail.com), [r.atkinson@cgiar.org](mailto:r.atkinson@cgiar.org)

^2^ Bioversity International, Rome, Italy, [c.kettle@cgiar.org](mailto:c.kettle@cgiar.org)

^3^ ETH Zürich, Institute of Terrestrial Ecosystems, Ecosystem Management, Zürich, Switzerland

*Corresponding author. Bioversity International, Av. La Molina 1895, CIP, Lima, Peru, P.O. Box 1558, Tel.: +51-1 3496017 ext 3108, Email: [evert.thomas@gmail.com](mailto:evert.thomas@gmail.com)

**
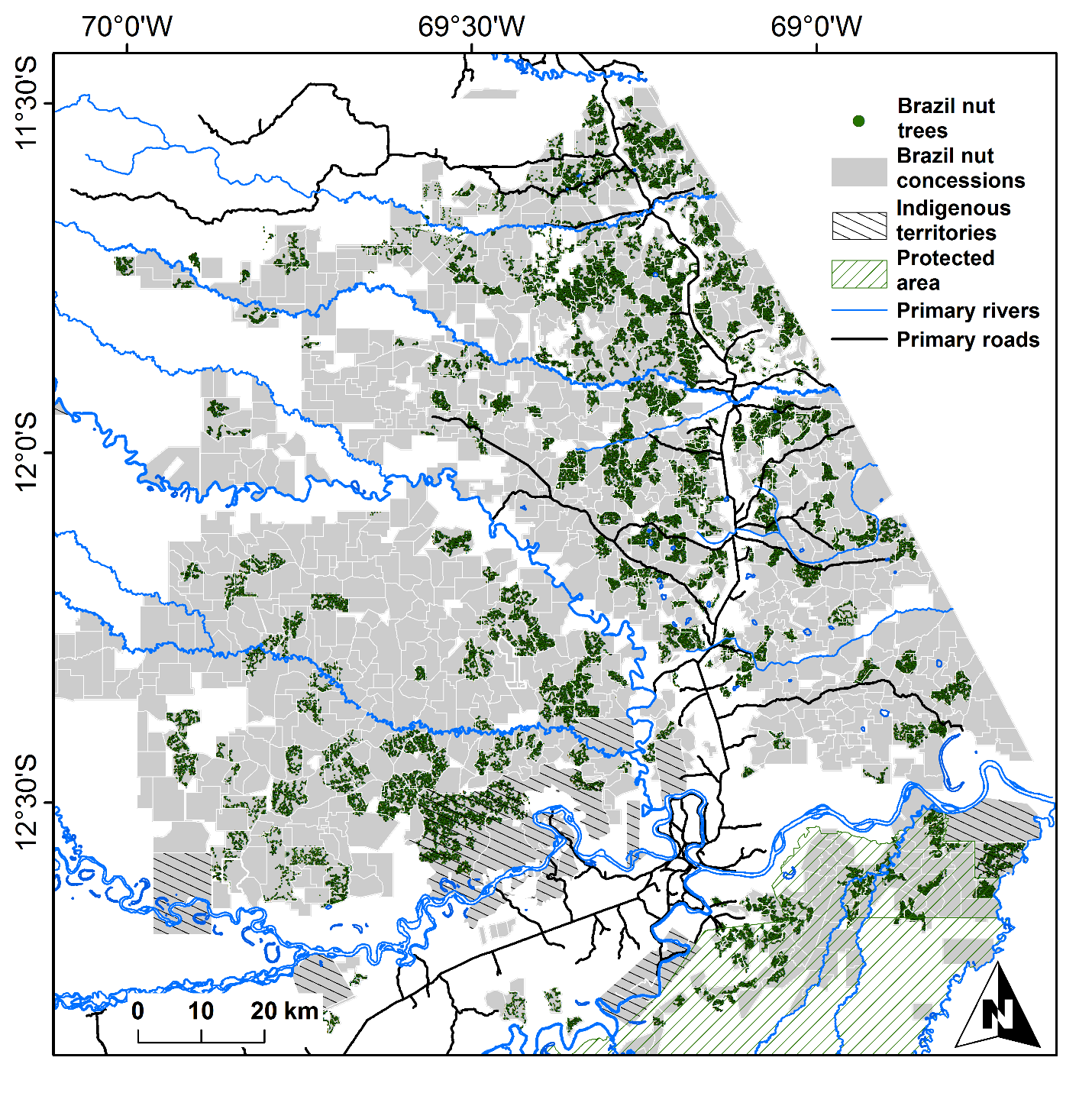
**

**Figure 1** Distribution of Brazil nut concessions in Madre de Dios, Peru, with approximate locations of the 135,528 individual Brazil nut trees (DBH≥10 cm) from 418 concessions considered in this paper.


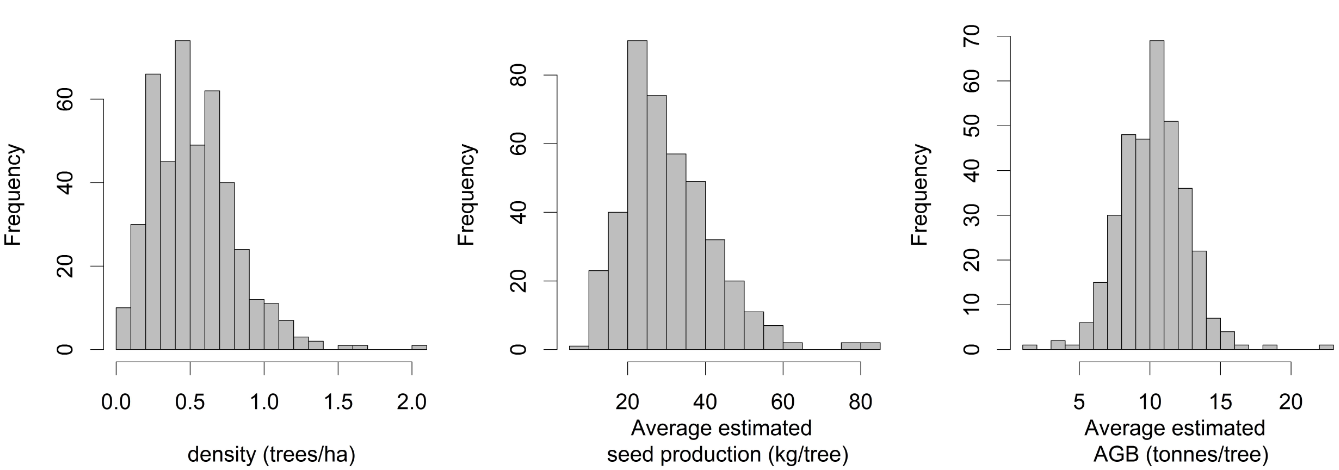


**Figure S2** Frequency diagrams of Brazil nut tree density, estimated seed production and AGB, averaged per concession.


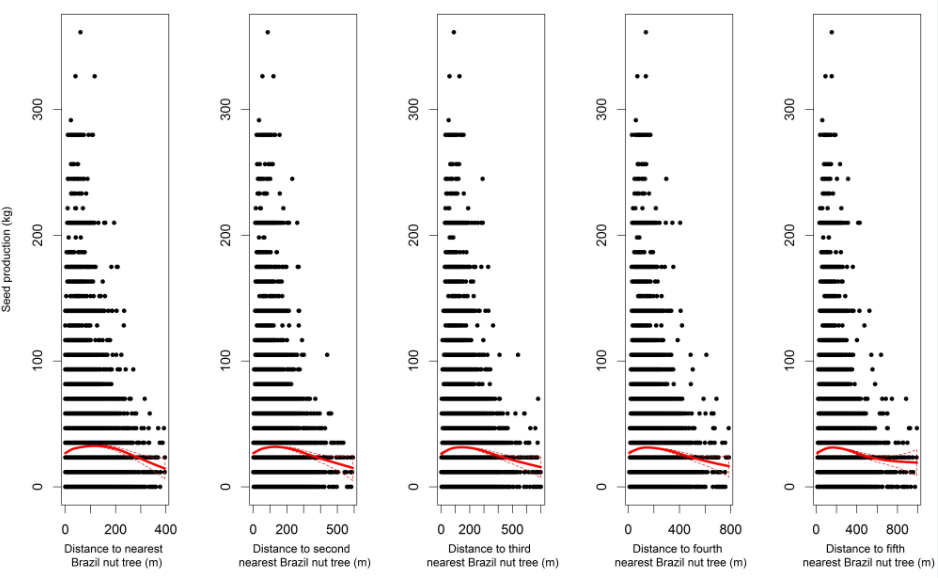


**Figure S3.** Relation between estimated seed production of Brazil nut trees and the distance to the five nearest conspecific trees. The solid red lines represent generalized adaptive model smoothers. Dashed lines show 95% confidence intervals. The first three figure are also included in Thomas et al^1^


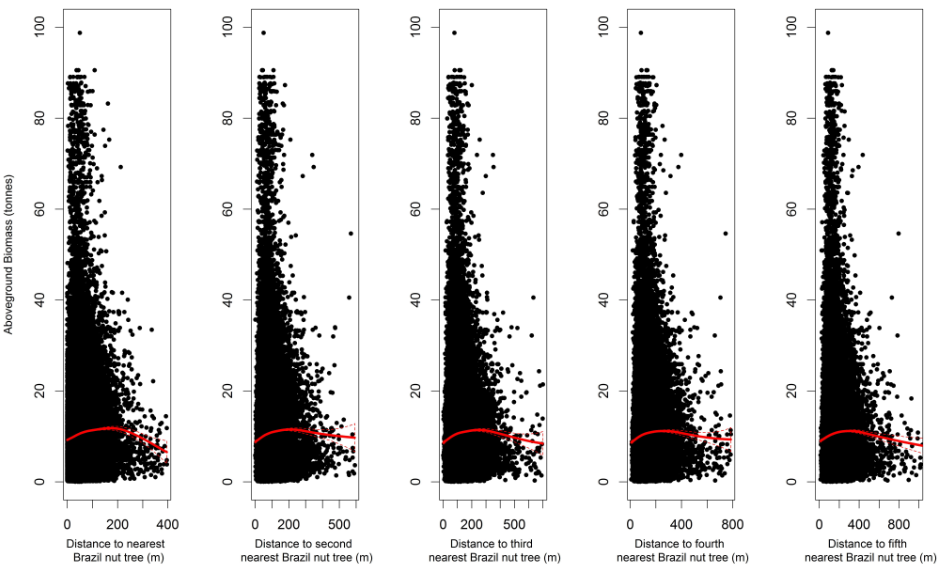


**Figure S4.** Relation between estimated AGB of Brazil nut trees and the distance to the five nearest conspecific trees. The solid red lines represent generalized adaptive model smoothers. Dashed lines show 95% confidence intervals.

**
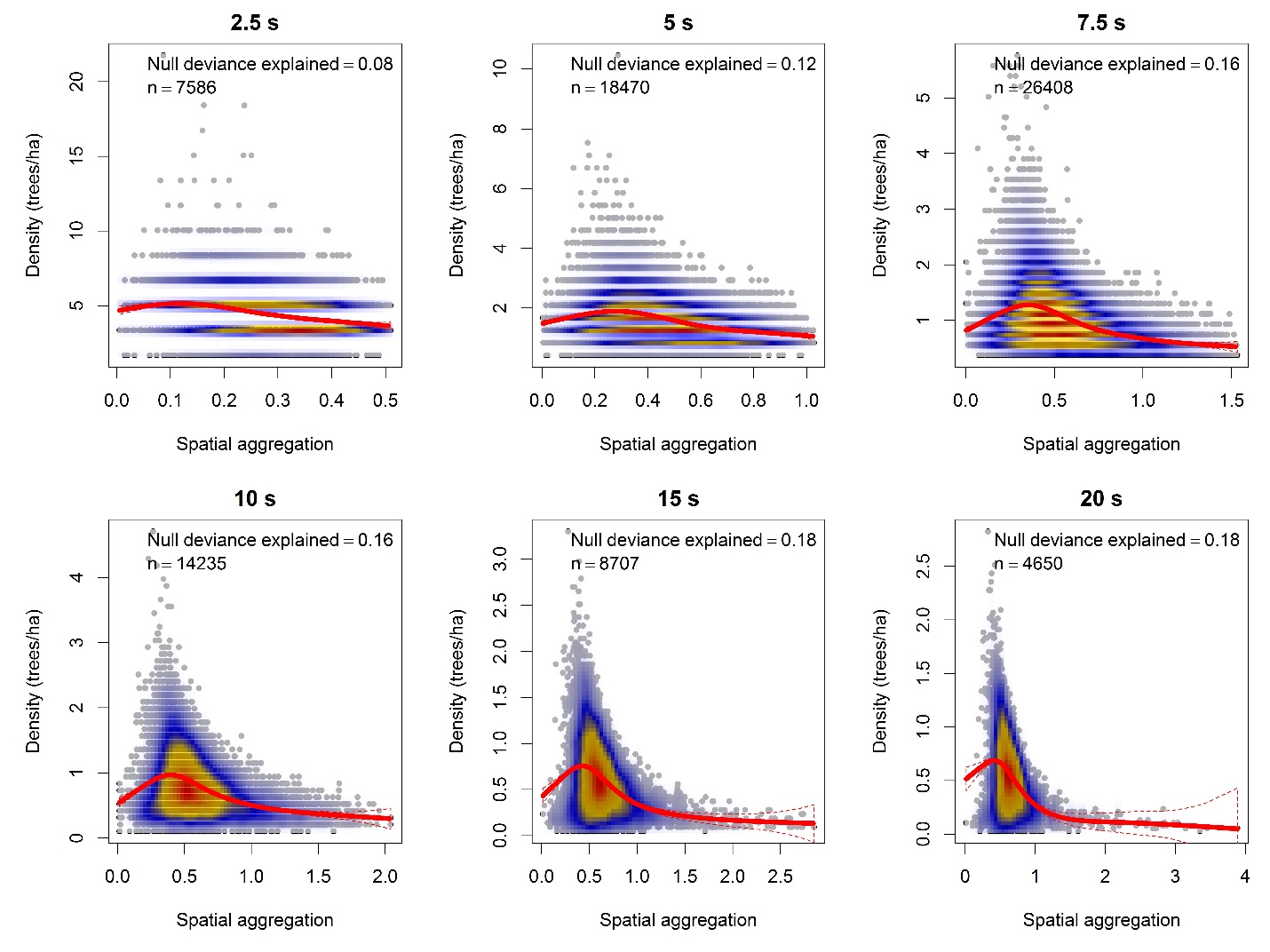
**

**Figure S5** Relations between spatial aggregation and density of Brazil nut trees for different grid cell sizes (expressed as arc seconds). An aggregation value of 1 is indicative for random patterns, more than 1 for regular (spaced-out) patterns, and less than 1 for aggregated patterns. Tree density values are rescaled to hectare unit. Numbers of grid cells with data for each spatial scale are indicated by *n*. Generalized adaptive model smoothers and 95% confidence intervals (solid and dashed red lines, respectively) are plotted on top of kernel density estimations (from high to low: red-yellow-blue-white).

**
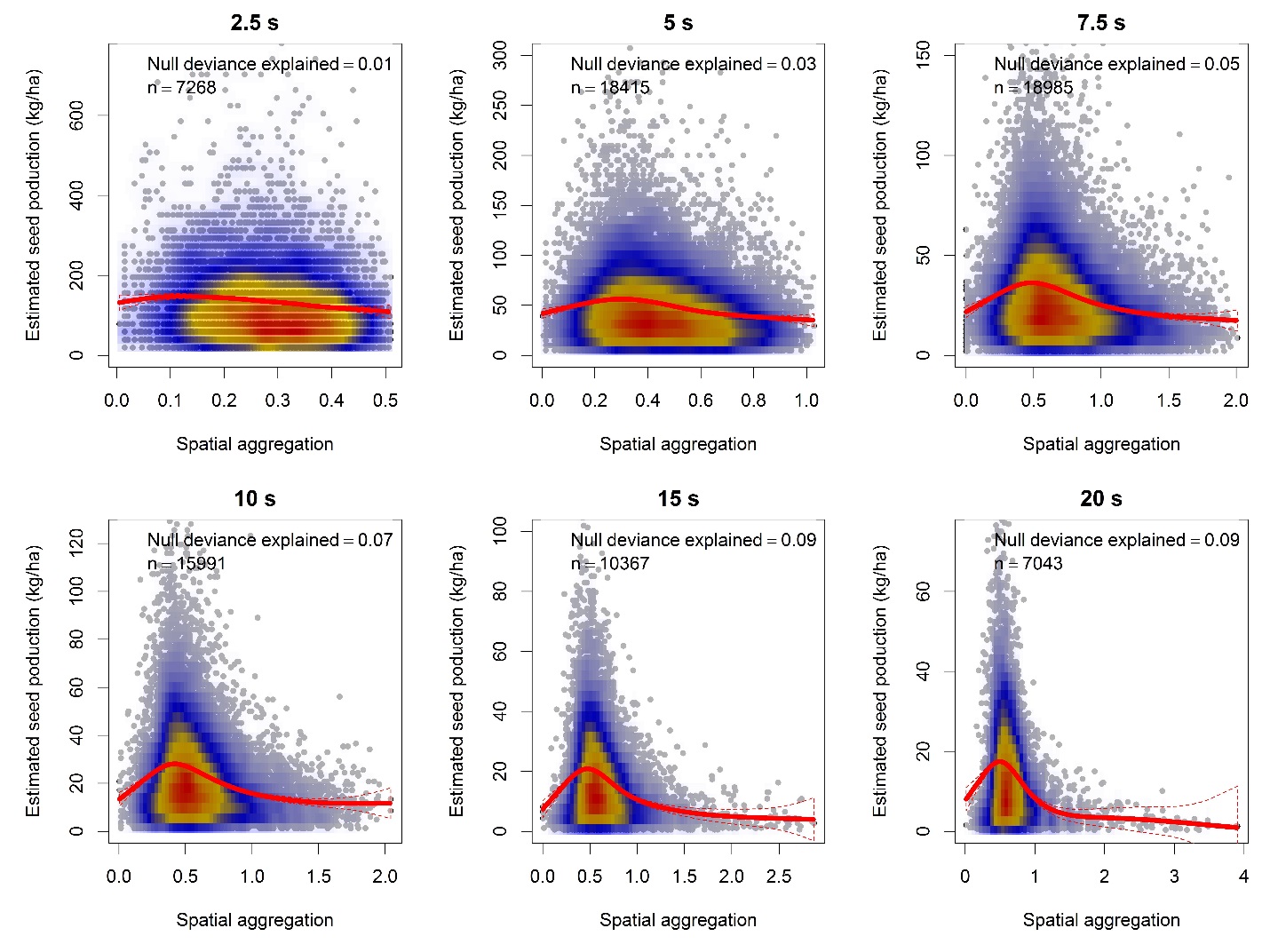
**

**Figure S6** Relations between spatial aggregation and area-based estimated seed production of Brazil nut for different grid cell sizes (expressed as arc seconds). An aggregation value of 1 is indicative for random patterns, more than 1 for regular (spaced-out) patterns, and less than 1 for aggregated patterns. Seed production values are rescaled to hectare unit. Numbers of grid cells with data for each spatial scale are indicated by *n*. Generalized adaptive model smoothers and 95% confidence intervals (solid and dashed red lines, respectively) are plotted on top of kernel density estimations (from high to low: red-yellow-blue-white).

**
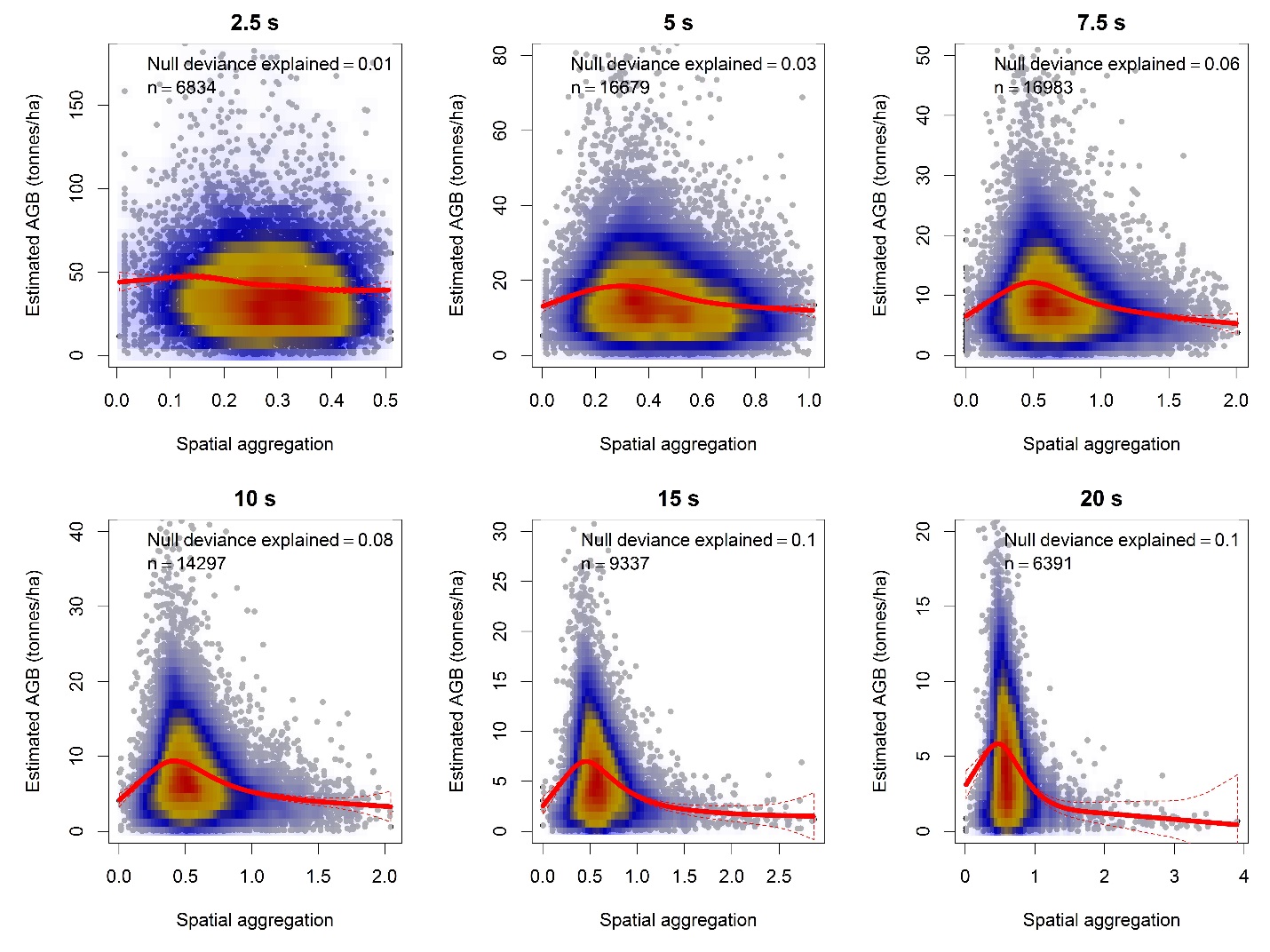
**

**Figure S7** Relations between spatial aggregation and area-based AGB of Brazil nut for different grid cell sizes (expressed as arc seconds). An aggregation value of 1 is indicative for random patterns, more than 1 for regular (spaced-out) patterns, and less than 1 for aggregated patterns. AGB values are rescaled to hectare unit. Numbers of grid cells with data for each spatial scale are indicated by *n*. Generalized adaptive model smoothers and 95% confidence intervals (solid and dashed red lines, respectively) are plotted on top of kernel density estimations (from high to low: red-yellow-blue-white).

**
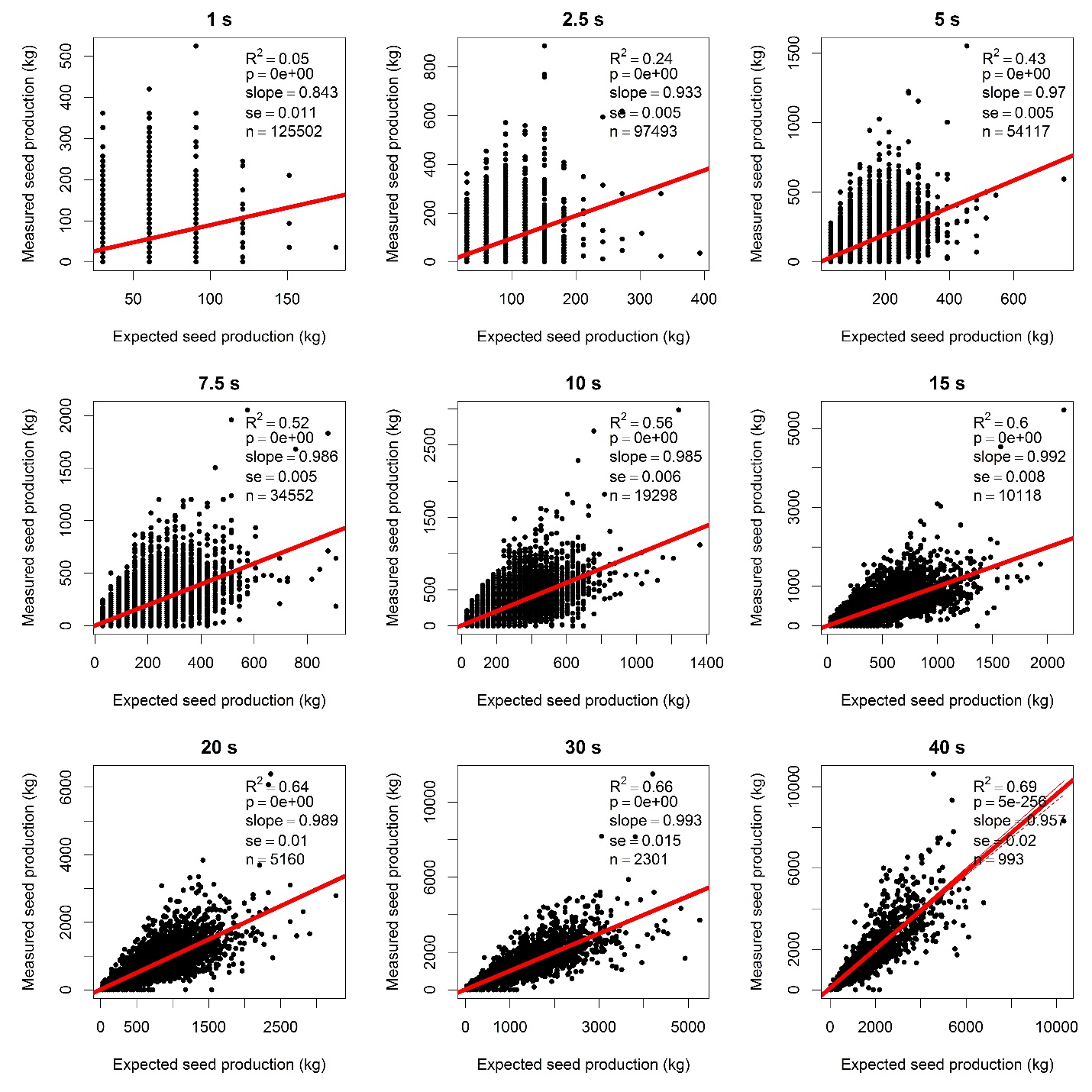
**

**Figure S8** Relations between expected and observed area-based seed production for different grid cell sizes (expressed as arc seconds). Expected values correspond with the number of trees per grid cells multiplied by the grand mean estimated seed production of 30.3kg per tree. Numbers of grid cells with data for each spatial scale are indicated by *n*. The solid red lines represent linear regression lines and the dashed lines show 95% confidence intervals.

**
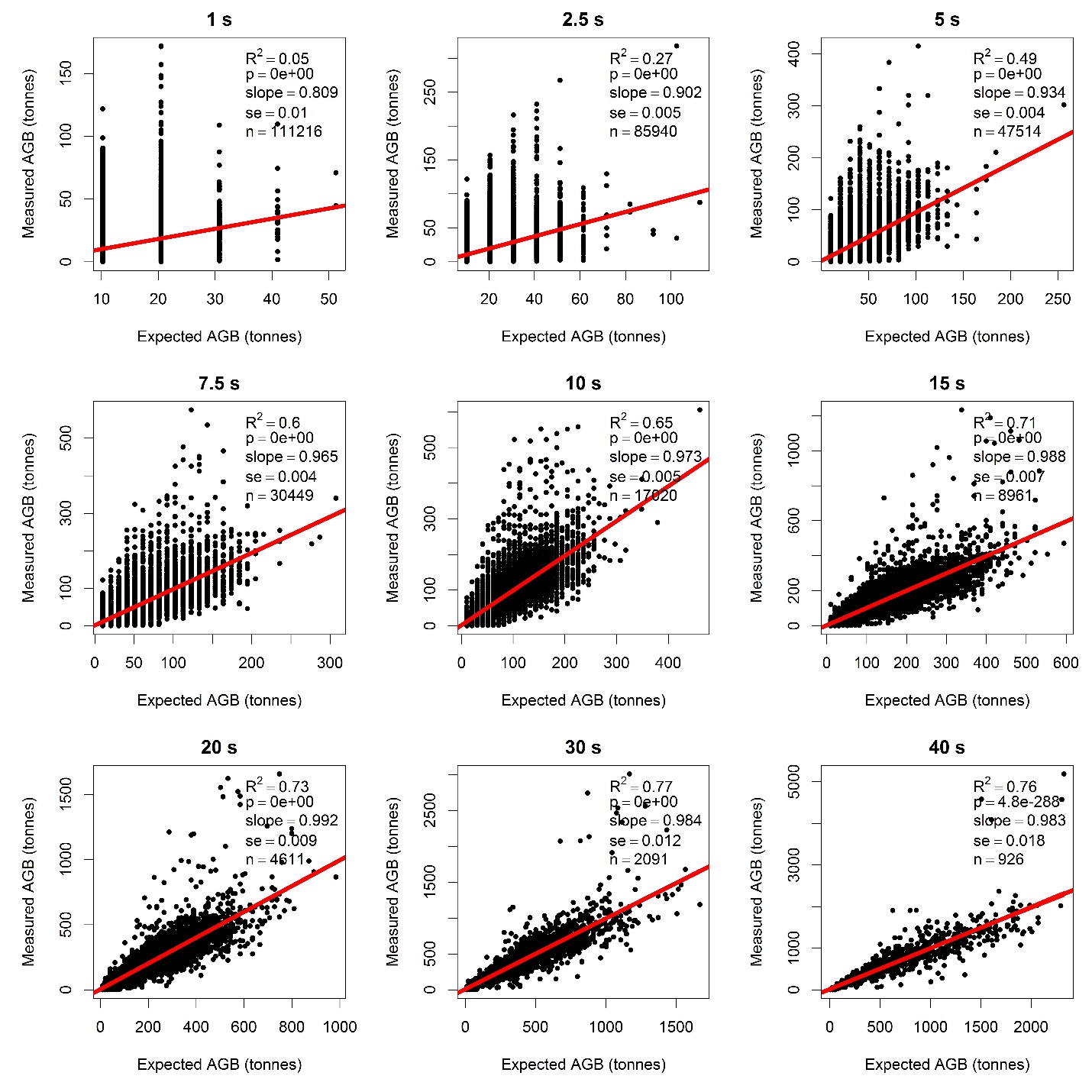
**

**Figure S9** Relations between expected and observed area-based AGB for different grid cell sizes (expressed as arc seconds). Expected values correspond with the number of trees per grid cells multiplied by the grand mean AGB of 10.25 tonnes per tree. Numbers of grid cells with data for each spatial scale are indicated by *n*. The solid red lines represent linear regression lines and the dashed lines show 95% confidence intervals.

**References**

1. Thomas, E., Valdivia, J., Alcázar Caicedo, Carolina Quaedvlieg, J., Wadt, L. H. O. & Corvera Gorminger, R. NTFP harvesters as citizen scientists: validating traditional and crowdsourced knowledge on seed production of Brazil nut trees in the Peruvian Amazon. *PlosOne* **8,** e0183743 (2017).
